# Inhibition of TRPA1 channel activity in sensory neurons by the glial cell line-derived neurotrophic factor family member, artemin

**DOI:** 10.1186/1744-8069-7-41

**Published:** 2011-05-27

**Authors:** Naoki Yoshida, Kimiko Kobayashi, Lina Yu, Shenglan Wang, Rengaowa Na, Satoshi Yamamoto, Koichi Noguchi, Yi Dai

**Affiliations:** 1 Department of Pharmacy, School of Pharmacy, Hyogo University of Health Sciences, Kobe, Hyogo 650-8530, Japan; 2 Department of Anatomy and Neuroscience, Hyogo College of Medicine, Nishinomiya, Hyogo 663-8501, Japan; 3 Department of physiology, Inner Mongolia Medical College, China

## Abstract

**Background:**

The transient receptor potential (TRP) channel subtype A1 (TRPA1) is known to be expressed on sensory neurons and respond to changes in temperature, pH and local application of certain noxious chemicals such as allyl isothiocyanate (AITC). Artemin is a neuronal survival and differentiation factor and belongs to the glial cell line-derived neurotrophic factor (GDNF) family. Both TRPA1 and artemin have been reported to be involved in pathological pain initiation and maintenance. In the present study, using whole-cell patch clamp recording technique, *in situ *hybridization and behavioral analyses, we examined the functional interaction between TRPA1 and artemin.

**Results:**

We found that 85.8 ± 1.9% of TRPA1-expressing neurons also expressed GDNF family receptor alpha 3 (GFR α3), and 87.5 ± 4.1% of GFRα3-expressing neurons were TRPA1-positive. In whole-cell patch clamp analysis, a short-term treatment of 100 ng/ml artemin significantly suppressed the AITC-induced TRPA1 currents. A concentration-response curve of AITC resulting from the effect of artemin showed that this inhibition did not change EC_50 _but did lower the AITC-induced maximum response. In addition, pre-treatment of artemin significantly suppressed the number of paw lifts induced by intraplantar injection of AITC, as well as the formalin-induced pain behaviors.

**Conclusions:**

These findings that a short-term application of artemin inhibits the TRPA1 channel's activity and the sequential pain behaviors suggest a role of artemin in regulation of sensory neurons.

## Background

Artemin belongs to glial cell line-derived neurotrophic factor (GDNF) family that regulates the development and the function of the nervous system. Artemin binds to the GFR α3/RET receptor complex and then activates several intracellular signaling pathways [1]. Artemin supports survival of sensory neurons *in vitro *and *in vivo *apparently by interacting with GFRα3, which is highly restricted in adult to neurons of the peripheral nervous system (sensory and sympathetic). GFRα3 is expressed by a subpopulation of nociceptive sensory neurons, some or all of which also express the Ret receptor tyrosine kinase, the transient receptor potential (TRP) ion channel proteins TRPV1 and TRPA1 [2,3]. The expression of these channels in GFR α3-positive neurons suggests that artemin signaling *via *GFR α3/Ret binding could modulate neuron sensitivity.

TRPA1 is a member of branch A of the TRP family of cation channels[4] and is expressed by a subset of small-sized DRG or trigeminal ganglia neurons in neonatal rats, adult rats and mice [5-7]. TRPA1 has been reported to be activated by various kinds of noxious compounds through covalent modification of cysteines [5,8-13]. TRPA1 is also activated by an endogenous aldehyde, 4-hydroxynonenal, bradykinin, intracellular pH and CO2 [8,14-16]. Studies using knockout mice demonstrated that TRPA1 is an important component of the transduction machinery through which environmental irritants and endogenous proalgesic agents depolarize nociceptors to elicit inflammatory pain [17,18]. A recent report showing the nearly complete attenuation of formalin-induced pain behaviors by pharmacological blockade or genetic ablation indicated that TRPA1 is crucial in inflammatory pain [19]. Taking the above into account, it is clear that this channel is importantly involved in pain transmission in the primary afferents and a potential target for analgesic development.

Recent reports suggested that artemin potentiates TRPV1 signaling and induces behavioral hyperalgesia. Overexpression of artemin increased the expression and sensitivity of TRPV1 and TRPA1 in trigeminal afferents signaling and induced behavioral hyperalgesia [20,21]. We have studied the modulation mechanism of TRPV1 and TRPA1 by inflammatory modulators and reported that trypsin/tryptase-PAR2 signaling or bradykinin-B2R signaling sensitizes TRPA1 channel through PLC and/or PKA pathways to induce inflammatory pain [22-24]. In the present study, we hypothesized that a functional interaction of artemin and TRPA1 might contribute to the pathogenesis of inflammatory pain. We observed high co-expression of the TRPA1 with the GFR α3 receptor and found a significant enhancement of desensitization of TRPA1 activity induced by artemin in rat DRG neurons, which was also confirmed at the behavioral level.

## Methods

### Immunohistochemistry

All animal experimental procedures were approved by the Hyogo University of Health Sciences Committee on Animal Research (#2008-05, #2008-10) and were performed in accordance with the National Institutes of Health guidelines on animal care. Adult male Sprague-Dawley (SD) rats (220-250 g; Japan Animals, Osaka, Japan) were perfused transcardially with 1% paraformaldehyde in 0.1 M phosphate buffer followed by 4% paraformaldehyde in 0.1 M phosphate buffer (PB, pH 7.4). The L4-5 DRGs were removed and processed for TRPA1 immunohistochemistry as described in our previous study [23]. For double immunofluorescence, tyramide signal amplification (TSA; NEN) fluorescence procedures were used for TRPA1 (1:10,000) staining. The raised rabbit primary antibody for TRPA1 [23] and biotin conjugated Griffonia simplicifolia Isolectin B4 (IB4, Sigma, St. Louis, MO) at 1:1000 combined with Alexa fluor 488 goat anti-rabbit IgG (1:500; Invitrogen/Molecular Probes, Inc., Carlsbad, CA) and strept-avidin conjugated with Alexa fluor 594 (1:500: Invitrogen/Molecular Probes), respectively, were used for double immunofluorescence staining [22].

### *In situ *hybridization histochemistry (ISHH)

Adult male SD rats weighing 200-250 g were killed by decapitation under deep ether anesthesia. The bilateral L4, L5 DRGs were dissected out, rapidly frozen in powdered dry ice, and cut on a cryostat at a 5 μm thickness. Sections were thaw mounted onto MAS-coated glass slides (Matsunami, Osaka, Japan) and fixed in 4% formaldehyde in 0.1 M PB (pH 7.4) for 20 min. After washing with PB, the sections were treated with 10 μg/ml protease K in 50 mM Tris/5 mM EDTA (pH 8.0) for 3 min at room temperature, postfixed in the same fixative, acetylated with acetic anhydride in 0.1 M triethanolamine, rinsed with PB, and dehydrated through an ascending ethanol series. For dual ISHH of TRPA1 and GDNF family receptors, we used a DIG-labeled probe for TRPA1 (GenBank accession number AY496961, nucleotides 302 - 769) and a ^35^S-labeled RNA probe for Ret (GenBank accession number U22513, nucleotides 13-319), GFR α1 (GenBank accession number U97142, nucleotides 652 - 955), or GFR α2 (GenBank accession number U97143, nucleotides 362 - 702), or GFR α3 (GenBank accession number AF184920, nucleotides 164 - 604) in the same sections, respectively. The details of the dual ISHH procedure have been described in our previous study [6].

### Mammalian cell culture

For primary culture of DRG neurons, DRGs were collected from the adult SD rats (100-200 g) using sterile techniques, and placed in ice-cold Earle's balanced salt solution (EBSS, Sigma). Adhering fat and connective tissue were removed and each DRG was placed immediately in a medium consisting of 2 ml of EBSS and 1.25 mg/ml of collagenase P (Sigma) and kept at 37°C for 60 min with occasional agitation. After dissociation of the DRG cells, this cell suspension was centrifuged for 5 min at 1000 rpm and the cell pellet was re-suspended in EBSS supplemented with 10% fetal bovine serum (FBS), 2 mM glutamax, penicillin, streptomycin and vitamin solution. Recombinant rat nerve growth factor (100 ng/ml, Sigma) was added to the medium.

### Electrophysiology

Whole-cell patch-clamp recordings were carried out at 1 day after dissociation of the DRG neurons. Voltage-clamp experiments were performed at -60 mV holding potential, and recordings were sampled at 5 kHz and filtered at 2 kHz. Current densities (pA/pF) and normalized currents (the third currents were normalized to the second currents evoked by an agonist) were measured. The current magnitude was quantified by peak current amplitude in all experiments. A normalized current was obtained by normalizing the third application-induced current to the second one, and just in case the magnitude of the second current by agonist was larger than a half of the first current to evaluate the results under less effect of the desensitization by the first current. In experiments with DRG neurons, after AITC (300 μM) application, capsaicin (10 μM) was applied at the end of recording to identify whether the AITC-induced current was mediated by TRPA1 channels. Data were obtained just in case the DRG neuron was sensitive to both AITC and capsaicin application, since an AITC-activated current in capsaicin-sensitive DRG neurons is certainly a TRPA1-mediated event [18]. The standard bath solution contained 140 mM NaCl, 5 mM KCl, 2 mM MgCl_2_, 2 mM CaCl_2_, 10 mM HEPES and 10 mM glucose, pH 7.4 (adjusted with NaOH). In some experiments, artemin (100 ng/ml) was included in the bath solutions. The pipette solution contained 140 mM KCl, 2 mM MgCl_2_, 0.5 mM CaCl_2_, 5 mM MgATP, 5 mM EGTA and 5 mM HEPES, pH 7.2 (adjusted with Tris-base). The concentration-response curves for the effect of artemin on AITC-induced current densities (pA/pF) were fit by the Hill equation using Origin 8.1 (OriginLab Corporation, Northampton, MA, USA). All patch-clamp experiments were performed at room temperature (~25°C; RT). The solutions containing drugs were applied to the chamber (180 μl) by a gravity system at a flow rate of 4-7 ml/min.

### Behavioral studies

Sixteen and 10 male adult SD rats (200-250 g) were used for the AITC-induced nocifensive behavioral analyses and formalin test, respectively. After adaptation, 50 μl artemin (10 μg/ml, in PBS) or PBS was injected intraplantarly into the rat left hind paw. Five minutes after these injections, rats received intradermal injection of 50 μl of AITC (3% in liquid paraffin, Wako Pure Chemical Industries, Ltd., Osaka, Japan) or formalin (3% in saline) to the same area of artemin-injected plantar surface. The rats were placed in a wire mesh cage immediately after the injection. The numbers of hind paw lifts and durations of flinches per 5 min interval during the initial 30 min post-injection of AITC and 60 min post-infection of formalin period were measured for the AITC-induced nocifensive behavior and formalin test, respectively. The total number of lifts and durations of flinches during the entire initial 30 min for AITC and 60 min for formalin post-injection were also calculated.

### Statistical analysis

All results are expressed as mean ± SEM. An unpaired t-test or ANOVA followed by Fisher's PLSD was used to compare the calcium imaging data and electrophysiological data between the groups. Two-way repeated ANOVA followed by Fisher's PLSD was applied to the behavioral data. A difference was accepted as significant if the probability was less than 5% (p < 0.05).

## Results

### Colocalization of TRPA1 with GDNF family receptors in rat DRG neurons

**Figure 1:**
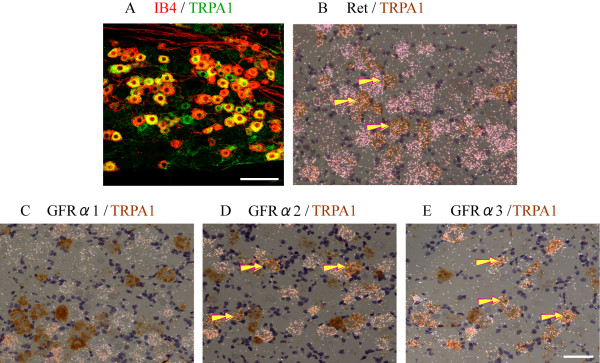
**Colocalization of TRPA1 and IB4, Ret or GFR αs in DRG neurons**. (A): Double immunostaining of TRPA1 (green) and IB4 (red) showed almost all of TRPA1 were expressed in IB4-labeled neurons of the naïve rat DRG. (B-E): Colocalization of TRPA1 mRNA (brown cells) and Ret (B), GFR α1 (C), GFR α2 (D), and GFR α3 (E) mRNAs (clusters of silver grains) using double ISHH with ^35^S-labeled antisense Ret and GFR αs probes combined with TRPA1 DIG-labeled probe. The bright field and dark field photographs are merged. Arrows indicate examples of double-labeled neurons. Scale bar = 100 μm in A, = 50 μm in B-D.

Nociceptors can be neurochemically classified into two subpopulations and modulated by neurotrophic factors differently in each population. Double immunofluorescent staining indicated that 82.2 ± 1.1% of TRPA1 positive neurons were also labeled with IB4, a marker of the nonpeptidergic and GDNF family members-dependent neurons, and 44.1 ± 1.9% of IB4-positive neurons were also stained for TRPA1 (Figure 1A). To further determine the histological evidence of colocalization with TRPA1 and GDNF family receptors, we examined the co-localization of TRPA1 mRNAs with Ret, GFR α1, GFR α2, and GFR α3 mRNAs using double ISHH (Figure 1B-E). We observed these mRNAs were expressed in 36.9 ± 2.6% (TRPA1), 77.4 ± 3.2% (Ret), 45.6 ± 4.9% (GFR α1), 44.1 ± 1.7% (GFR α2) and 38.4 ± 2.7% (GFR α3) of the total neuronal profiles. A significant population of TRPA1-positive neurons were also labeled for c-Ret and GFR α3 (arrows in Figure 1B, E) in the DRG (Table 1). The high percentages of colocalization; 99.3 ± 0.6% and 85.8 ± 1.9% of TRPA1 expressing neurons were Ret- and GFR α3-positive, respectively, suggest that TRPA1 is selectively expressed by artemin-sensitive Ret/GFR α3 neurons (Figure 1B and 1E). In contrast to the high incidence of co-localization of TRPA1 and GFR α3, only 13.5 ± 7.7% and 45.9 ± 7.6% of TRPA1-positive neurons were also labeled for GFR α1 and GFR α2, respectively (Figure 1C and 1D). This high percentage of co-expression of TRPA1 with GFR α3 in DRG neurons indicates a possible interaction between them in primary afferent neurons.

**Table 1:** The percentage of DRG profiles that co-express IB4, Ret, GFR α1, GFR α2, or GFR α3 and TRPA1 mRNA (mean ± SE)

x	x/total	TRPA1/x	x/TRPA1
TRPA1	36.9 ± 2.6%	100%	100%

IB4	N.A.	44.1 ± 1.9%	82.2 ± 1.1%

GFR α1	45.6 ± 4.9%	10.2 ± 5.1%	13.5 ± 7.7%

GFR α2	44.1 ± 1.7%	37.8 ± 7.0%	45.9 ± 7.6%

GFR α3	38.4 ± 2.7%	87.5 ± 4.1%	85.8 ± 1.9%

Ret	77.4 ± 3.2%	47.5 ± 5.5%	99.3 ± 0.6%

### Artemin suppresses AITC-induced current in DRG neurons

Based on the histological results, we hypothesized that artemin may play important roles in the functional modulation of TRPA1. To this end, we performed whole-cell voltage-clamp experiments in cultured rat DRG neurons. We found that application of artemin alone to cause no change of membrane currents in cultured DRG neurons. AITC, as a selective activator of TRPA1, had been used in patch-clamp experiment to detect the TRPA1 current. A previous study indicates that there are still AITC-sensitive cells in TRPA1 knockout mice, but these are not the TRPV1-expressing cells where most TRPA1 is expressed [18]. To confirm the AITC-evoked current in DRG is a TRPA1-mediated event, capsaicin at 10 μM was applied at the end of recording using the patch-clamp preparation (see Materials and Methods section).

**Figure 2:**
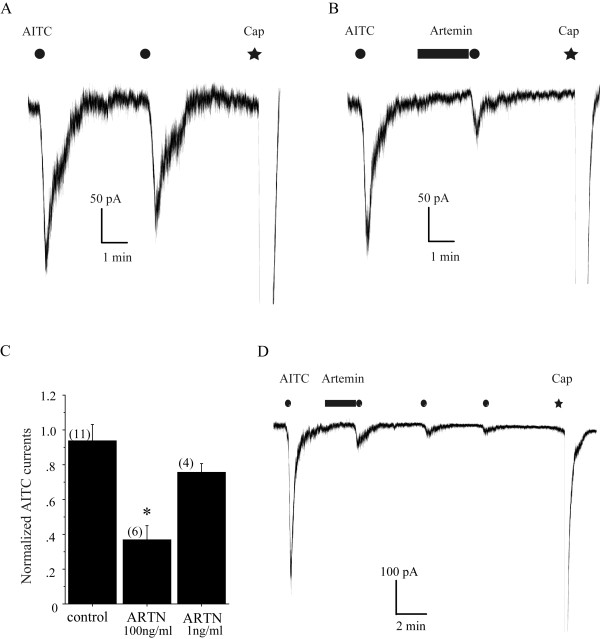
**A short-term treatment of artemin decreased AITC-induced currents in DRG neurons**. (A, B): Representative traces show TRPA1 currents produced by repeated AITC application without (A) or with (B) artemin (100 ng/ml) treatment. AITC was re-applied after artemin administration for 2 min. (C): Bar graph shows the effect of artemin on AITC activated currents. Currents were normalized to the values evoked initially by AITC application in the absence of artemin. Numbers in parenthesis indicate cells tested. (D): Representative traces show time-course of TRPA1 currents induced by repeated AITC application after artemin (100 ng/ml) treatment. AITC (300 μM) in a bath solution was perfused for 20s in all experiments.

**Figure 3:**
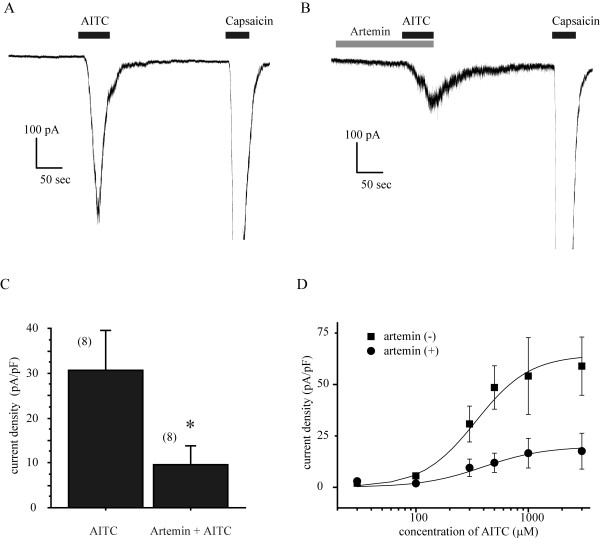
**Suppression of AITC-induced current density in DRG neurons by artemin**. (A, B): Representative traces show TRPA1 currents produced by AITC application (300 μM, 1 min) in the absence (A) or presence (B) of artemin (100 ng/ml). AITC was applied for 1 min after artemin administration for 2 min. (C): Bar graph shows the effect of artemin on AITC activated current density (pA/pF). Numbers in parenthesis indicate cells tested. * p < 0.05 versus control (absence of artemin), unpaired t-test. (D): Concentration-response curves for AITC-induced current in the present or absence of artemin. Figure shows averaged data fitted with the Hill equation (see methods). EC50 = 343 μM and Maximum current density = -64.4 pA/pF in the absence of artemin, EC50 = 396 μM and Maximum current density = -19.8 pA/pF in the presence of artemin. Data for each point were generated from separate neurons by application of AITC for 1 min after pretreatment of artemin or vehicle. Holding potential (Vh) = -60 mV in all experiments.

We observed that the AITC-activated inward currents in DRG neurons underwent a weak tachphylaxis, giving a smaller response on repeated applications of 300 μM AITC for 20 sec (Figure 2A). We have previously demonstrated that G protein-coupled receptor signals can potentiate the TRPA1 activity [23,24]. However, in the present study, instead of potentiation, after 2 min pretreatment with artemin (100 ng/ml), reapplication of AITC with the same dose produced much smaller current responses than the preceding application of AITC. The normalized currents were significantly smaller in the artemin-pretreated group than that in the control group, which was not pretreated with artemin (0.9 ± 0.09-fold change, n = 11 for control; 0.3 ± 0.09-fold change, n = 6 for artemin; p < 0.005). A smaller effect was detected in cells pretreated with artemin in low concentrations (1 ng/ml) (0.8 ± 0.05-fold change, n = 4 for 1 ng/ml p > 0.05 versus control) (Figure 2B and 2C). This suppression effect lasted at least 8 min after artemin application (Figure 2D). To further confirm the inhibitory effect of TRPA1 by artemin, we also tested the effect of artemin on AITC-induced current density in DRG neurons. After 2 min pretreatment of artemin, the current density induced by AITC (300 μM, 1 min) was significantly suppressed (-30.8 ± 8.74 pA/pF, n = 8 for control, -9.5 ± 4.25 pA/pF, n = 8 for artemin, p < 0.05 vs control) (Figure 3A-C). To examine how artemin changes TRPA1 responsiveness, we measured AITC-induced current density by applying a range of concentrations of AITC in the absence or presence of artemin. We found that treatment of artemin suppressed the maximum response (-64.4 ± 13.36 pA/pF for control vs -19.8 ± 11.16 pA/pF for artemin) without conspicuously affecting EC_50 _(343 μM for control vs 396 μM for artemin) (Figure 3D). These data clearly indicate a suppressive effect of artemin on TRPA1 channel activity.

### Artemin suppressed TRPA1-mediated pain behaviors

**Figure 4:**
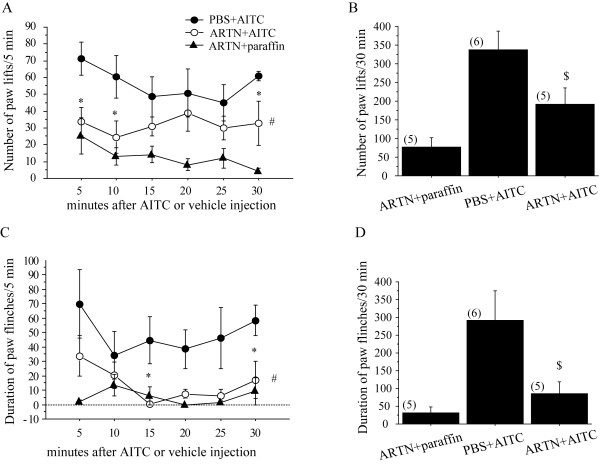
**Artemin suppressed nocifensive behaviors induced by AITC intraplantar injection**. Artemin (10 μg/ml) or PBS was pre-treated subcutaneously 5 min before AITC (3% in 50 μl liquid paraffin) injection. (A-D): Left panels show the time course of the number of hind paw liftings (A) and the duration of paw flinches (C) after AITC injection with artemin (ARTN) or PBS pre-treatment. The number counted per 5 min interval in the initial 30 min post-injection period. Right panels show cumulative number of paw lifts (B) and cumulative duration of flinches (D) over the first 30 min period after injection of AITC. * p < 0.05; versus PBS + AITC at each time point (unpaired t-test), # p < 0.05; ## p < 0.001; versus PBS + AITC group (two-way repeated ANOVA followed by Fisher's PLSD), $ p < 0.05; $$ p < 0.01; versus PBS + AITC group (two-way factorial ANOVA). Numbers in parentheses indicate number of rats used in each group.

**Figure 5:**
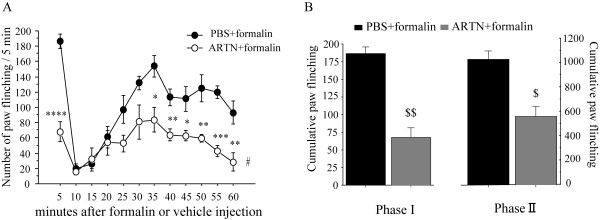
**Artemin suppressed nocifensive behaviors induced by formalin**. Artemin (10 μg/ml) or PBS was pre-treated subcutaneously 5 min before formalin (3% in 50 μl saline) injection. Time course (A) and cumulative data (B) of the number of hind paw flinches after formalin injection with artemin or PBS pre-treatment were measured. The number counted per 5 min interval in the initial 1 h post-injection period in A. B shows cumulative number of paw flinches during the Phase I (0-5 min) and the second Phase II (10-60 min) after injection of formalin. * p < 0.05; ** p < 0.01; *** p < 0.0005; **** p < 0.0001 versus PBS + formalin (unpaired t-test), # p < 0.001; versus PBS + formalin (two-way repeated ANOVA followed by Fisher's PLSD), $ p < 0.005; $$ p < 0.0001 versus PBS + formalin (two-way factorial ANOVA). Numbers in parentheses indicate number of rats used in each group.

Activation of TRPA1 by pungent natural products suggests a nociceptive role for TRPA1. Both AITC and formalin has been reported to cause nocifensive behaviors in animals through TRPA1 activation [19,23]. To test whether artemin treatment could suppress TRPA1-mediated pain behaviors, we performed intraplantar injections with AITC or formalin after pretreatment of artemin (10 μg/ml) and recorded nocifensive behaviors of rats. Consistent with our previous studies [23-25], both AITC- and formalin-injection induced a significant lifting and flinching behavior of the injected hindpaw during the 30 or 60 min post-injection period, whereas such behaviors were not observed in vehicle-injected rats (data not shown). The injection of artemin did not cause any inflammatory reactions (e.g. redness, swelling) and acute nocifensive behavior (e.g. paw lifting, flinching or licking). Five min after pre-treatment with the artemin, AITC or formalin was injected into the same area of the hind paw. Pre-treatment with artemin induced a significant decrease of the number of paw lifts and the duration of paw flinches in the initial 30 min post-injection period of AITC, compared to those of rats pre-treated with vehicle (Figure 4). Administration of artemin preceding the formalin injection also reduced the number of flinches observed in both Phase I (0-5 min) and Phase II (10-60 min) (Figure 5).

## Discussion

In recent years, the role of artemin, a GDNF family member, in mediating neuropathic and inflammatory pain has been received much attention. Artemin is known to be one of the survival factors for sensory and sympathetic neurons *in vitro *and *in vivo. *The long-term intrathecal or systemic administration of artemin prevented many of the nerve injury-induced changes in the histochemistry of nociceptor neurons, and produced dose- and time-related reversal of nerve injury-induced pain behaviors [26-28]. On the other hand, overexpression of artemin up-regulated expression of TRPV1 and TRPA1 channels and subsequently led to an increase of neuronal activity and hyperalgesia [2,20]. Acute application of artemin induced a significant potentiation of TRPV1 function and produced acute thermal hyperalgesia [21]. In the present study, in contrast to the potentiation of TRPV1 function by artemin, we observed that a short-term application of artemin significantly suppressed TRPA1 channels activity and the TRPA1-mediated pain behaviors.

Artemin binds to GFR α3/Ret to induce extracellular signals [1]. We found TRPA1 was highly coexpressed with GFR α3 and RET (Figure 1). This finding is consistent with a previous report that indicates nearly all GFR α3-positive neurons express TRPV1 immunoreactivity, and most of these neurons are also TRPA1 positive [2]. The high co-localization of TRPA1 and GFR α3 provides a possible histological prerequisite of the functional interaction between these two molecules.

We found in the present study that a short term application of artemin significant reduced the AITC-induced TRPA1 current. This inhibitory regulation was examined in two ways. First, artemin was delivered between two applications of AITC and then the normalized current (the next AITC-current versus the first one) was collected (Figure 2). This approach allowed us to confirm that the tested neuron was indeed a TRPA1-expressing one, but tachyphylaxis of AITC-induced current may have interrupted the analysis and made it difficult to explain the reduced normalized current solely as an inhibition of TRPA1 channels, rather a cooperative effect of an artemin-induced inhibition and the tachyphylaxis may have taken place. Therefore, we also performed an experiment with sensory neurons pretreated by artemin and then applied AITC to evaluate the current density. Both of these results clearly indicated inhibitions of TRPA1 channel function by acute application of artemin.

Acute application of artemin has been reported to induce potentiation of another TRP channel, TRPV1, and produce acute thermal hyperalgesia [21], TRPV1 and TRPA1 are structurally endowed with the same TRP domain but have distinctly different intracellular loops. AITC activates TRPA1 by covalently modifying cysteine residues located in the N terminus of the channel, different from a classical lock-and-key binding mechanism for capsaicin-TRPV1. Thus it is not a wonder that the regulatory mechanism of TRPV1 and TRPA1 may differ. For example, PKC has been reported to potentiate TRPV1 [22,29,30] but did not have any effect on TRPA1[23,24]. On the other hand, Elitt et al have reported that overexpression of artemin up-regulated expression of TRPV1 and TRPA1 channels and subsequently led to greater neuronal activity and hyperalgesia [2,20]. These reports suggested a post-transcriptional regulation of artemin (which might contribute as a neurotrophic factor) on TRPV1 and TRPA1 channels, are not discordant with our findings.

TRPA1 is known to be expressed on sensory neurons and acted as an important component of pain. If artemin can suppress the TRPA1activation, pain sensation that is caused through the TRPA1 channel may also be suppressed by the artemin activation. Topical application of AITC has been reported to excite sensory nerve fibers, thereby producing acute pain [5,17,31]. Consistent with our electrophysiological data, artemin produced a significantly persistent suppression of the duration of paw flinch and the number of paw lifts induced by intraplantar injection of AITC (Figure 3). A recent study indicates that TRPA1 is the principal site of formalin's pain-producing action in rodents [19]. The formalin test is a widely used model of continuous pain resulting from formalin-induced tissue injury. Subcutaneous injection of formalin into the rat hindpaw produces a biphasic pain response that consists of an early, acute phase (Phase I) and a late, tonic phase (Phase II) that is manifested behaviorally as lifting, flinching or licking of the affected paw, and these behaviors are robust and readily quantifiable [32,33]. Phase I is thought to result from direct activation of primary afferent sensory neurons, whereas Phase II has been proposed reflect the combined effects of afferent input and central sensitization in the dorsal horn. In our present study, we found artemin treatment significantly suppressed the formalin-induced pain behaviors at both two phases (Figure 3). Direct inhibition of TRPA1 activity by local injection of artemin in the plantar nerve terminal may result the suppression of behaviors in Phases I, then the reduced input of primary afferent may contribute the reduction of pain behaviors in Phase II. These findings suggest that a short-term application of artemin inhibits the TRPA1 channel's activity and the sequential pain behaviors.

The mechanism of artemin-induced inhibition of TRPA1 activity is not clear. Two potential mechanisms are suggested to be involved in this regulation. One would be that artemin increases EC_50 _by suppressing the binding affinity of AITC to TRPA1. The other mechanism would be that artemin lowers the channel density of TRPA1 on cell membranes through promoting receptor internalization or by inhibiting membrane insertion of TRPA1 channels. In the present study, the concentration-response curves of AITC resulting from the effect of artemin (Figure 3C) revealed that artemin lowered the maximal response of AITC without conspicuously altering EC_50_, indicating that artemin may suppress AITC response in DRG neurons principally by lowering the channel density. Distinct constitutive and regulated vesicular trafficking mechanisms have critical roles not only in controlling the surface expression of TRP channels but also their activation in response to stimuli [34]. TRPA1 may cycle between the plasma membrane and intracellular compartments, and the balance between membrane insertion and retrieval determines its surface abundance and activity [35]. A recent report indicated that TRPA1 channel desensitization in sensory neurons was regulated by internalization of itself [36]. Artemin-induced GFR α3/RET activation results in stimulation of multiple signal transduction pathways, including the MAP kinase/Erk and PI3 kinase/Akt pathways. A potential mechanism of inhibition of TRPA1 by artemin in the present study may be the internalization of the channels, which is regulated by the downstream of GFR α3/RET intracellular signals. On the other hand, it has been reported that TRPA1 is functionally inactivated in sensory neuron by extracellular Ca^2 ^influx [37]. Although activation of GFR α3/RET signals results in intracellular Ca^2 ^mobilization, however, it is not a likely mechanism for the AITC-evoked current decrease observed in our experiment. The reason simply comes from that cytosolic-free Ca^2 ^is tightly chelated with the 5 mM EGTA included in the pipette solution. Anyway, detailed mechanisms of artemin regulation on TRPA1 need to be further studied.

## Conclusions

Artemin is widely expressed in nervous system [38], supports the survival and regulates the differentiation of peripheral neurons, including sympathetic, parasympathetic, sensory and enteric neurons [3,39-41]. We now show the interaction of artemin and TRPA1 in the rat nervous system and provide a novel role of artemin in inhibitory regulation of sensory neurons in the current study. Artemin may have certain functions in both the neurotrophic effects and inhibitory actions on sensory neurons.

## Competing interests

The authors declare that they have no competing interests.

## Authors' contributions

NY carried out the electrophysiological and behavioral studies, performed the statistical analysis, and participated in drafting the manuscript. KK carried out the histochemistry studies. LY, GN, SW and SY participated in the electrophysiological studies. SW also participated in drafting the manuscript. KN supervised the project and edited the manuscript. YD conceived of the project, designed and coordinated the studies, and drafted the manuscript. All authors contributed to data interpretation, have read and approved the final manuscript.
